# Construction and validation of a simple, scoreable model for predicting infection risk in patients with multiple myeloma: a retrospective single-center study

**DOI:** 10.3389/fonc.2025.1672927

**Published:** 2026-01-02

**Authors:** Sheng-ke Tu, Jing Yang, Sha-dong Min, Hong-jie Fan, Mimi Hu, Juan Tian, Min Li, Kui Song

**Affiliations:** 1 Department of Hematology, The First Affiliated Hospital of Jishou University, Jishou, Hunan, China; 2 Department of Pharmacy, The First Affiliated Hospital of Jishou University, Jishou, Hunan, China

**Keywords:** multiple myeloma, infection, scoring model, risk factors, predicting infection

## Abstract

**Objective:**

This study aims to identify the risk factors for infection in patients with multiple myeloma (MM) and to develop a predictive model for infection.

**Methods:**

We retrospectively analyzed the clinical data of 180 multiple myeloma patients with MM who underwent chemotherapy at the First Affiliated Hospital of Jishou University from January 2017 to December 2022. A predictive model for infection events was constructed based on these data.

**Results:**

In the modeling group, 34 out of 90 patients (37.78%) experienced infections, whereas in the validation group, 40 out of 90 patients (44.44%) had infections. Binary logistic regression analysis showed that the levels of C-reactive protein level, fasting blood glucose level, lactate dehydrogenase level, Eastern Cooperative Oncology Group (ECOG) score, and the percentage of bone marrow plasma cell percentage were independent risk factors for infection in patients with MM (P < 0.05). The infection prediction model developed using these variables demonstrated good accuracy, with an area under the ROC curve of 0.827 (95% CI: 73.66%–91.78%) in the modeling group and for the validation group being 0.760 (95% CI: 65.97%–85.93%) in the validation group.

**Conclusion:**

This study confirms that C-reactive protein level, fasting blood glucose level, lactate dehydrogenase level, ECOG score, and the percentage of bone marrow plasma cell percentage are significant risk factors for infection in patients with MM.

**Clinical significance:**

This infection prediction model offers substantial clinical value by enabling a shift from reactive management to proactive, preventive intervention for infections in this vulnerable population.

## Highlights

- In recent years, the overall survival of patients with multiple myeloma (MM) patients has improved significantly due to the emergence of proteasome inhibitors, immunosuppressants, monoclonal antibodies, and hematopoietic stem cell transplantation. However, the compromised immune dysfunction caused by these treatments further increases the risk of infections in MM patients with MM, thereby escalating their treatment burden and potentially interrupting planned therapeutic regimens. Numerous studies have identified infections as a leading cause of mortality in MM patients with MM, particularly in those with early-stage disease, underscoring the need for early prevention and timely intervention.
- Given the rising incidence of infections and infection-related mortality, the ability to predict infection events holds substantial clinical value during the treatment of MM treatment. Early identification and prevention of such events can markedly reduce mortality rates. Nevertheless, existing prediction models remain limited, and no standardized guidelines are currently available. Therefore, this study retrospectively analyzed infection-related risk factors for infections in MM patients with MM and developed a predictive model for infection events in this population.
- This study retrospectively examined the clinical data of 180 MM patients with MM and identified infection-related risk factors using regression analysis. A predictive model for infection events was subsequently developed, demonstrating meaningful utility for early risk prediction and early intervention. At present, there is a the lack of relevant research, both domestically and internationally.
- This study identified five key predictors of infection in multiple myeloma patients with MM: C-reactive protein, fasting blood glucose, lactate dehydrogenase, Eastern Cooperative Oncology Group (ECOG) score, and bone marrow plasma cell percentage. Leveraging these variables, the new model provides a practical and effective tool for forecasting infections and facilitating early clinical interventions.

## 1 Introduction

Multiple myeloma (MM) is a malignant hematological tumor that predominantly affects middle-aged and elderly individuals. It is primarily characterized by the clonal proliferation of plasma cells in the bone marrow, the secretion of monoclonal immunoglobulin or light chain fragments, and the presence of “CRAB” symptoms, including hypercalcemia, renal impairment, anemia, and bone destruction. MM accounts for 1% of all cancers and approximately 10% of all hematologic malignancies (1, 2). In recent years, the introduction of proteasome inhibitors, immunosuppressants, monoclonal antibodies, and hematopoietic stem cell transplantation has significantly improved the overall survival of multiple myeloma (MM) patients with MM. However, the immunosuppressive effects associated with these therapies increase susceptibility to infection, thereby elevating the treatment burden and potentially interrupting planned therapeutic regimens (3, 4). Numerous studies have identified infection as a major cause of death in MM patients with MM, particularly in those newly diagnosed with the disease (5–8), highlighting the need for early prevention and timely intervention.

Several studies, both domestic and international, have summarized and analyzed infection-related risk factors in patients with MM. Pulmonary infections in multiple myeloma (MM) patients. These studies have been reported as the most common type. Infections occur throughout all stages of treatment and present substantial challenges to controlling nosocomial infections (9, 10). The heightened susceptibility of MM patients with MM to infection is complex and multifactorial, potentially resulting from impaired immune function combined with treatment-related immunosuppressants agents, such as lenalidomide and thalidomide. Given the increasing incidence of infections and infection-related mortality, the ability to predict infection events carries important clinical significance during MM treatment. Early identification and prevention could reduce mortality.

However, available prediction models remain relatively limited, and no unified standard currently exists. Therefore, this study retrospectively analyzed infection-related risk factors in patients with MM and constructed a predictive model for infection events in this population.

## 2 Cases and methods

### 2.1 Diagnostic criteria and cases of MM

According to the diagnostic criteria of the National Comprehensive Cancer Network (NCCN) and the International Myeloma Working Group(IMWG) (11, 12), 180 patients diagnosed with multiple myeloma (MM at the First Affiliated Hospital of Jishou University from January 2017 to December 2022 were enrolled in this single-center, retrospective analysis.

Patients who received immunoglobulin replacement therapy, pneumococcal vaccination, or antibiotic prophylaxis; underwent hematopoietic stem cell transplantation; did not receive chemotherapy; or were diagnosed with asymptomatic (smoldering) MM were excluded. Patient data—including sex, age, disease duration, Eastern Cooperative Oncology Group (ECOG) score, neutrophil count, lymphocyte count, C-reactive protein level, fasting blood glucose level, serum creatinine level, lactate dehydrogenase level, immunotherapy, and bone marrow plasma cell percentage—were obtained from the electronic medical record system.

Informed consent was obtained from all patients or from their immediate family members. Written informed consent was obtained from the patient and his guardians for the publication of this study. The study was conducted in accordance with the modified Helsinki Declaration and was approved by the ethics committee of the First Affiliated Hospital of Jishou University (EC-LCKY2023038).

### 2.2 Diagnostic criteria for infection

The diagnosis of infection was based on the “Diagnostic Criteria for Nosocomial Infection (trial*)* issued by the Ministry of Health (China) and the Centers for Disease Control and Prevention (CDC).

The diagnostic criteria for pulmonary infection were as follows:

1. the patient exhibited cough, thick sputum, and moist rales in the lungs, along with one of the following:
  ① fever;
  ② an increased white blood cell count and/or neutrophil ratio;
  ③ evidence of inflammatory infiltration on chest X-ray.
2. patients with stable chronic airway diseases (chronic bronchitis with or without obstructive emphysema, bronchiectasis, or asthma) who developed secondary acute infection, with etiological changes or new or clearly worsened chest X-ray findings compared with those at admission.

### 2.3 Statistical analysis

SPSS 26.0 software was used for data processing. Measurement data were expressed as mean ± standard deviation, and the independent-samples t-test was applied to compare patients with and without infection in the modeling group. Univariate logistic regression was used to identify infection-related variables. Count data were expressed as percentages and analyzed using the chi-square test. Multivariate logistic regression analysis was performed to determine independent risk factors. A value of P < 0.05 was considered statistically significant.

## 3 Research results

### 3.1 Clinical characteristics of the disease

Among the 180 patients included in this study, 98 (54.44%) were male and 82 (45.56%) were female. The largest age group was 60–75 years, comprising 123 patients (68.33%) (**Table 1A**). A total of 74 infections occurred (34 in the modeling group and 40 in the validation group), with an overall infection rate of 41.11%. The general data and clinical characteristics of the 180 MM patients with MM are shown in **Table 1A**. Contingency analysis of infection among patients with MM is presented in **Table 1B**.

**Table 1A:** General data and clinical features of the 180 MM patients.

Clinical features	Classification	Frequency	Percentage (%)
Sex	Male	82	45.56
	Female	98	54.44
Age (year)	≥65	172	95.56
	<65	8	4.44
Number of neutrophils (/L)	0.5–1.5 × 10^9^	20	11.11
	<0.5 × 10^9^	2	1.11
	>1.5 × 10^9^	158	87.78
Lymphocyte count (/L)	0.8–4 × 10^9^	122	67.78
	<0.8 × 10^9^	40	22.22
	>4 × 10^9^	18	10
C-reactive protein levels (mg/L)	≥10	66	36.67
	<10	114	63.33
Blood glucose levels (mmol/L)	≥6.1	48	26.67
	<6.1	132	73.33
Lactate dehydrogenase levels (U/L)	≥250	22	12.22
	<250	158	87.78
Bone marrow plasma cells (n, %)	≥30%	16	8.89
	<30%	164	91.11
ECOG	1	17	9.44
	2	52	28.89
	3	64	35.56
	4	47	26.11
M-protein types (n, %)	IgA	89	49.44
	IgD	6	3.33
	IgG	69	38.33
	k free light chain	11	6.11
	λ free light chain	5	2.78
ISS stage (n, %)	III	120	66.67
	II	55	30.56
	I	5	2.78
Durie–Salmon stage (n, %)	III A	102	56.67
	III B	55	30.56
	II A	13	7.22
	II B	10	5.56
Whether immunotherapy was administered	No	88	48.89
	Yes	92	51.11
Immunotherapy type	No	82	45.56
	Lenalidomide	24	13.33
	Thalidomide	73	40.56
	Potomalidomide	1	0.56
Whether to relapse	No	171	95
	Yes	9	5
Presence of any catheter	No	171	95
	Yes	9	5
Duration of the disease	≥6 months	138	76.67
	<6 months	42	23.33
Chemotherapy regimen	Contains lenalidomide but without bortezomib	18	10
	Contains bortezomib without lenalidomide	132	73.33
	Neither bortezomib nor lenalidomide was included	4	2.22
	Contains both bortezomib and lenalidomide	26	14.44
Immunotherapy type	1 cycle	78	43.33
	2 cycles	30	16.67
	3 cycles	36	20
	4 cycles	9	5
	5 cycles	9	5
	6 cycles	2	1.11
	7 cycles	5	2.78
	8 cycles	5	2.78
	>8 cycles	6	3.33
Total		180	100

**Table 1B:** Univariate analysis of infection in MM patients.

Clinical features	Classification	Infection	Total	χ^2^	*p*
Sex	Male	36 (45.00)	82 (45.56)	0.018	0.894
	Female	44 (55.00)	98 (54.44)		
Total		80	180		
Age (year)	≥65	72 (90.00)	172 (95.56)	10.465	0.001**
	<65	8 (10.00)	8 (4.44)		
Total		80	180		
Number of neutrophils (/L)	0.5–1.5 × 10^9^	8 (10.00)	20 (11.11)	1.841	0.398
	<0.5 × 10^9^	0 (0.00)	2 (1.11)		
	>1.5 × 10^9^	72 (90.00)	158 (87.78)		
Total		80	180		
Lymphocyte count (/L)	0.8–4 × 10^9^	44 (55.00)	122 (67.78)	25.974	0.000**
	<0.8 × 10^9^	18 (22.50)	40 (22.22)		
	>4 × 10^9^	18 (22.50)	18 (10.00)		
Total		80	180		
C-reactive protein levels (mg/L)	≥10	42 (52.50)	66 (36.67)	15.545	0.000**
	<10	38 (47.50)	114 (63.33)		
Total		80	180		
Blood glucose levels (mmol/L)	≥6.1	33 (41.25)	48 (26.67)	15.661	0.000**
	<6.1	47 (58.75)	132 (73.33)		
Total		80	180		
Lactate dehydrogenase levels (U/L)	≥250	14 (17.50)	22 (12.22)	3.739	0.053
	<250	66 (82.50)	158 (87.78)		
Total		80	180		
Bone marrow plasma cells (n, %)	≥30%	12 (15.00)	16 (8.89)	6.64	0.010**
	<30%	68 (85.00)	164 (91.11)		
Total		80	180		
ECOG	1	2 (2.50)	17 (9.44)	8.657	0.034*
	2	27 (33.75)	52 (28.89)		
	3	29 (36.25)	64 (35.56)		
	4	22 (27.50)	47 (26.11)		
Total		80	180		
M-protein types (n, %)	IgA	34 (42.50)	89 (49.44)	5.96	0.202
	IgD	2 (2.50)	6 (3.33)		
	IgG	34 (42.50)	69 (38.33)		
	K free light chain	8 (10.00)	11 (6.11)		
	λ free light chain	2 (2.50)	5 (2.78)		
Total		80	180		
ISS stage (n, %)	III	57 (71.25)	120 (66.67)	2.398	0.301
	II	20 (25.00)	55 (30.56)		
	I	3 (3.75)	5 (2.78)		
Total		80	180		
Durie–Salmon stage (n, %)	III A	42 (52.50)	102 (56.67)	3.337	0.343
	III B	28 (35.00)	55 (30.56)		
	II A	4 (5.00)	13 (7.22)		
	II B	6 (7.50)	10 (5.56)		
Total		80	180		
Whether immunotherapy was administered	No	42 (52.50)	88 (48.89)	0.751	0.386
	Yes	38 (47.50)	92 (51.11)		
Total		80	180		
Immunotherapy type	No	36 (45.00)	82 (45.56)	5.524	0.137
	Lenalidomide	15 (18.75)	24 (13.33)		
	Thalidomide	28 (35.00)	73 (40.56)		
	Potomalidomide	1 (1.25)	1 (0.56)		
Total		80	180		
Whether to relapse	No	75 (93.75)	171 (95.00)	0.474	0.491
	Yes	5 (6.25)	9 (5.00)		
Total		80	180		
Presence of any catheter	No	74 (92.50)	171 (95.00)	1.895	0.169
	Yes	6 (7.50)	9 (5.00)		
Total		80	180		
Duration of the disease	≥6 months	64 (80.00)	138 (76.67)	0.894	0.344
	<6 months	16 (20.00)	42 (23.33)		
Total		80	180		
Chemotherapy regimen	Contains lenalidomide but without bortezomib	6 (7.50)	18 (10.00)	6.31	0.097
	Contains bortezomib without lenalidomide	57 (71.25)	132 (73.33)		
	Neither bortezomib nor lenalidomide was included	4 (5.00)	4 (2.22)		
	Contains both bortezomib and lenalidomide	13 (16.25)	26 (14.44)		
Total		80	180		
Immunotherapy type	1 cycle	29 (36.25)	78 (43.33)	11.132	0.194
	2 cycles	21 (26.25)	30 (16.67)		
	3 cycles	14 (17.50)	36 (20.00)		
	4 cycles	4 (5.00)	9 (5.00)		
	5 cycles	3 (3.75)	9 (5.00)		
	6 cycles	1 (1.25)	2 (1.11)		
	7 cycles	3 (3.75)	5 (2.78)		
	8 cycles	2 (2.50)	5 (2.78)		
	>8 cycles	3 (3.75)	6 (3.33)		
Total		80	180		

### 3.2 Study on infection risk factors

The t-test results in the modeling group with and without infection showed that there were significant differences in C-reactive protein level, fasting blood glucose level, lactate dehydrogenase level, bone marrow plasma cell percentage, and ECOG score between patients with and without infection (P<0.05), as shown in **Table 1**. Binary logistic regression analysis indicated that these same variables were independent risk factors for infection in patients with MM (P < 0.05), as shown in **Table 2**. Univariate analysis findings for infection in MM patients with MM in the modeling group are shown in **Table 3**.

**Table 2:** T-test analysis results of the modeling group MM with and without infection.

Independent risk factors	Infection or not (mean ± standard deviation)		*t*	*p*
	0.0 (*n* = 56)	1.0 (*n* = 34)		
Lymphocyte count (/L)	1.26 ± 0.70	9.50 ± 14.08	-3.41	0.002**
C-reactive protein levels (mg/L)	9.26 ± 11.25	40.50 ± 67.66	-2.631	0.013*
Blood glucose levels (mmol/L)	5.10 ± 1.03	6.32 ± 1.12	-5.156	0.000**
Lactate dehydrogenase levels (U/L)	162.80 ± 42.37	219.18 ± 95.46	-3.254	0.002**
Bone marrow plasma cells (%)	4.32 ± 16.07	20.36 ± 21.49	-3.761	0.000**
ECOG	2.45 ± 0.83	2.97 ± 0.80	-2.949	0.004**

*p<0.05 **p<0.01.

**Table 3:** Univariate logistic regression analysis of MM infection in the modeling group.

Influencing factor	Regression coefficient	Standard error	*z*	Wald χ2	*p*	OR	OR 95% CI
C-reactive protein levels(mg/L)	0.964	0.379	2.545	6.476	0.011	2.622	1.248–5.510
Blood glucose levels(mmol/L)	0.087	0.028	3.085	9.517	0.002	1.091	1.032–1.152
Lactate dehydrogenase levels(U/L)	1.063	0.386	2.753	7.580	0.006	2.896	1.358–6.172
Bone marrow plasma cells(%)	0.006	0.006	1.067	1.138	0.286	1.006	0.995–1.017
ECOG score	-0.018	0.021	-0.848	0.720	0.396	0.982	0.943–1.024
Intercept	2.031	0.755	2.690	7.236	0.007	7.620	1.735–33.460

**Table 4:** Univariate analysis of infection in MM patients in the modeling group.

Clinical features	Classification	Infection	Total	χ2	*p*
Sex	Male	11 (32.35)	40 (44.44)	3.236	0.072
	Female	23 (67.65)	50 (55.56)		
Total		34	90		
Age (year)	≥65	30 (88.24)	86 (95.56)	6.895	0.009**
	<65	4 (11.76)	4 (4.44)		
Total		34	90		
Number of neutrophils (/L)	0.5–1.5 × 10^9^	5 (14.71)	13 (14.44)	0.614	0.736
	<0.5 × 10^9^	0 (0.00)	1 (1.11)		
	>1.5 × 10^9^	29 (85.29)	76 (84.44)		
Total		34	90		
Lymphocyte count (/L)	0.8–4 × 10^9^	15 (44.12)	56 (62.22)	17.722	0.000**
	<0.8 × 10^9^	8 (23.53)	22 (24.44)		
	>4 × 10^9^	11 (32.35)	12 (13.33)		
Total		34	90		
C-reactive protein levels (mg/L)	≥10	20 (58.82)	32 (35.56)	12.911	0.000**
	<10	14 (41.18)	58 (64.44)		
Total		34	90		
Blood glucose levels (mmol/L)	≥6.1	17 (50.00)	23 (25.56)	17.162	0.000**
	<6.1	17 (50.00)	67 (74.44)		
Total		34	90		
Lactate dehydrogenase levels (U/L)	≥250	7 (20.59)	9 (10.00)	6.807	0.009**
	<250	27 (79.41)	81 (90.00)		
Total		34	90		
Bone marrow plasma cells (n, %)	≥30%	9 (26.47)	11 (12.22)	10.34	0.001**
	<30%	25 (73.53)	79 (87.78)		
Total		34	90		
ECOG	1	0 (0.00)	7 (7.78)	9.859	0.020*
	2	11 (32.35)	33 (36.67)		
	3	13 (38.24)	35 (38.89)		
	4	10 (29.41)	15 (16.67)		
Total		34	90		
M-protein types (n, %)	IgA	14 (41.18)	43 (47.78)	1.314	0.859
	IgD	1 (2.94)	3 (3.33)		
	IgG	15 (44.12)	35 (38.89)		
	K free light chain	3 (8.82)	6 (6.67)		
	λ free light chain	1 (2.94)	3 (3.33)		
Total		34	90		
ISS stage (n, %)	III	25 (73.53)	60 (66.67)	3.065	0.216
	II	7 (20.59)	27 (30.00)		
	I	2 (5.88)	3 (3.33)		
Total		34	90		
Durie–Salmon stage (n, %)	III A	20 (58.82)	52 (57.78)	0.834	0.841
	III B	11 (32.35)	27 (30.00)		
	II A	2 (5.88)	6 (6.67)		
	II B	1 (2.94)	5 (5.56)		
Total		34	90		
Whether immunotherapy was administered	No	16 (47.06)	44 (48.89)	0.073	0.787
	Yes	18 (52.94)	46 (51.11)		
Total		34	90		
Immunotherapy type	No	14 (41.18)	38 (42.22)	0.046	0.977
	Lenalidomide	6 (17.65)	15 (16.67)		
	Pomalidomide	14 (41.18)	37 (41.11)		
Total		34	90		
Whether to relapse	No	33 (97.06)	86 (95.56)	0.291	0.59
	Yes	1 (2.94)	4 (4.44)		
Total		34	90		
Presence of any catheter	No	32 (94.12)	87 (96.67)	1.102	0.294
	Yes	2 (5.88)	3 (3.33)		
Total		34	90		
Duration of the disease	≥6	29 (85.29)	69 (76.67)	2.274	0.132
	<6	5 (14.71)	21 (23.33)		
Total		34	90		
Chemotherapy regimen	Contains lenalidomide but without bortezomib	6 (17.65)	14 (15.56)	4.636	0.201
	Contains bortezomib without lenalidomide	24 (70.59)	71 (78.89)		
	Neither bortezomib nor lenalidomide was included	1 (2.94)	1 (1.11)		
	Contains both bortezomib and lenalidomide	3 (8.82)	4 (4.44)		
Total		34	90		
Immunotherapy type	1 cycle	17 (50.00)	43 (47.78)	9.03	0.34
	2 cycles	6 (17.65)	9 (10.00)		
	3 cycles	3 (8.82)	17 (18.89)		
	4 cycles	2 (5.88)	4 (4.44)		
	5 cycles	3 (8.82)	5 (5.56)		
	6 cycles	0 (0.00)	2 (2.22)		
	7 cycles	1 (2.94)	3 (3.33)		
	8 cycles	1 (2.94)	4 (4.44)		
	>8 cycles	1 (2.94)	3 (3.33)		
Total		34	90		

* *p*<0.05 ** *p*<0.01.

## 4 Model construction and verification

### 4.1 Data grouping

Based on a 1:1 ratio of 1:1, 180 patients were randomly divided into a modeling group and a validation group. The modeling group included 90 patients, of whom 34 had infections (infection rate: 37.78%). The validation group also included 90 patients, of whom 40 had infections (infection rate: 44.44%).

### 4.2 Model building

According to the partial regression coefficients of each risk factor identified in the logistic regression analysis (**Table 4**), a prediction scoring model for nosocomial infection in MM patients with MM was constructed. The scoring criteria were as follows:

- C-reactive protein > 10 mg/L
- fasting blood glucose > 6.1 mmol/L
- bone marrow plasma cell percentage ≥ 30%
- lactate dehydrogenase ≥ 245 U/L
- ECOG score > 3

These five risk factors were assigned scores of 2 points, 1 point, 1 point, 2 points, and 1 point, respectively (**Table 5**). According to the prediction scoring system, the infection rates of patients with total scores of 0–2 points, 3–5 points, and 6–7 points were 44.12%, 47.06%, and 8.82%, respectively (**Table 6**).

**Table 5:** Variable assignment for logistic regression analysis.

Variable	Assignment
C-reactive protein level > 10mg/L	2
Blood glucose levels > 6.1mmol/L	1
Lactate dehydrogenase level ≥ 245U/L	2
Percentage of bone marrow plasma cells ≥ 30%	1
ECOG score > 3	1

**Table 6:** Risk stratification of infected patients in the modeling group.

Risk level	Risk stratification	Presence or absence of infection		Total
		Absence	Presence	
Risk level	0–2	51 (91.07)	15 (44.12)	66 (73.33)
	3–5	3 (5.36)	16 (47.06)	19 (21.11)
	6–7	2 (3.57)	3 (8.82)	5 (5.56)
total		56	34	90

**Figure 1:**
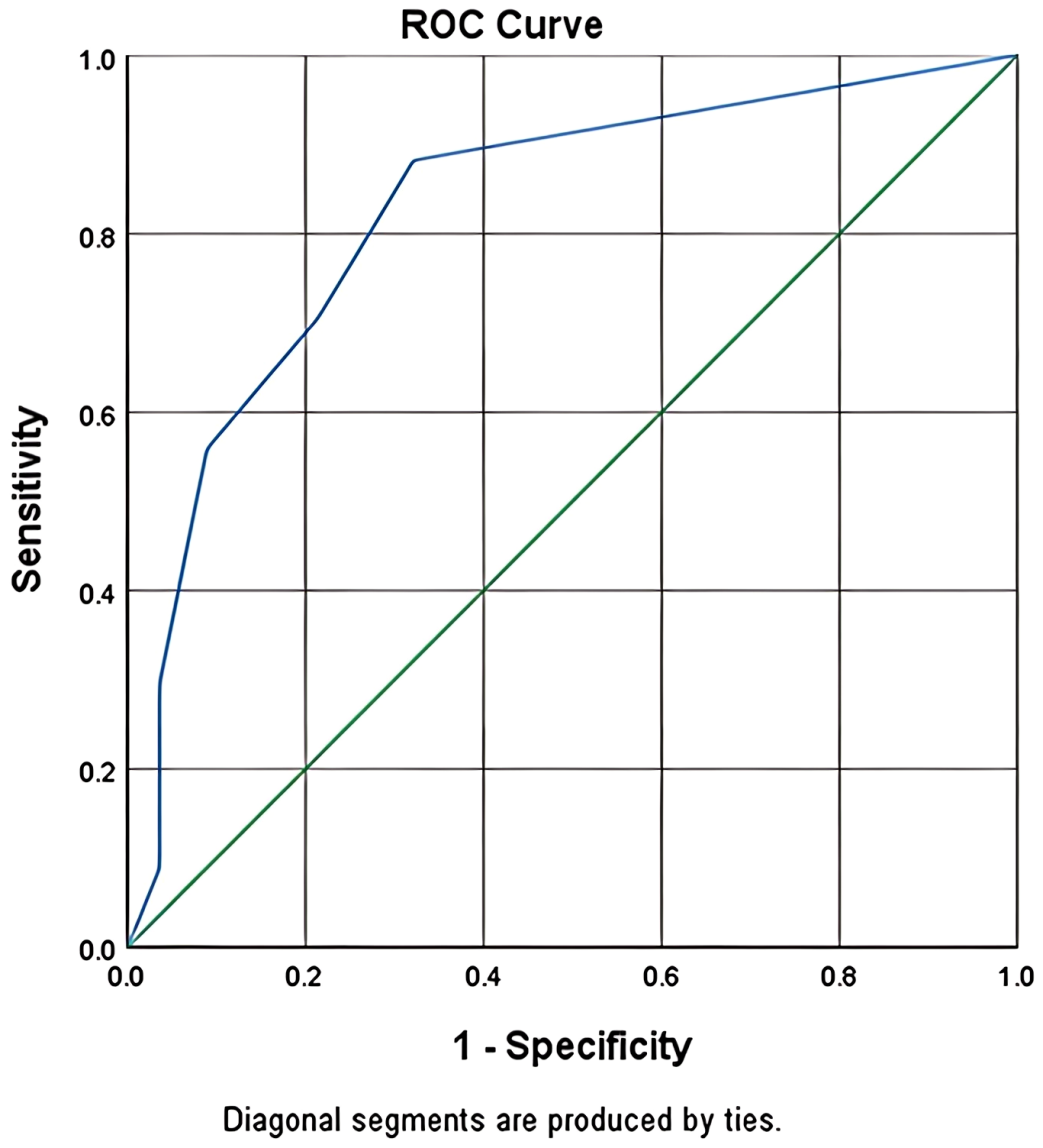
For the modeling group, the ROC curve was used to calculate the specificity and sensitivity, and the results showed that the AUC value corresponding to the modeling was 0.827(95% CI:73.66%~91.78%).

In the modeling group, the ROC curve was used to calculate the specificity and sensitivity. The area under the curve (AUC) was 0.827 (95% CI: 73.66%–91.78%) (see **Figure 1**). These findings indicate that the infection risk assessment model developed in this study demonstrated good discriminative ability for the data of the modeling dataset.

### 4.3 Model verification

**Figure 2:**
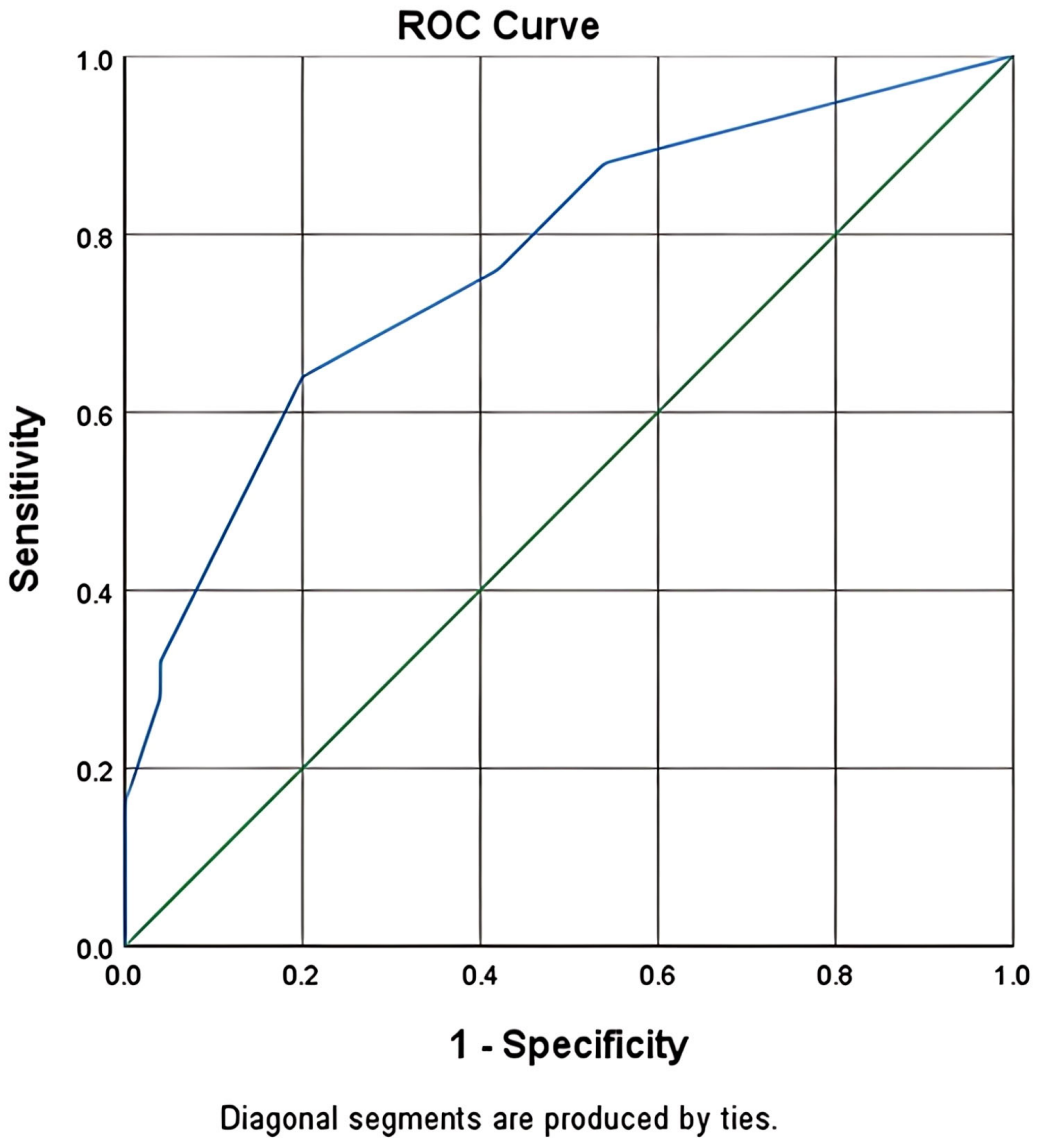
For the validation group, ROC curve was used to calculate the specificity and sensitivity, and the results showed that the AUC corresponding to the model was 0.760 (95%CI: 65.97%-85.93%).

The data of MM patients in the validation group was used to further assess model performance. The infection rates in patients with scores of 0–2 points, 3–5 points, and 6–7 points were 45%, 45%, and 10%, respectively (**Table 7**). The results showed that the AUC for the validation group was 0.760 (95% CI: 65.97%–85.93%) (**Figure 2**). These results demonstrate that the infection risk assessment model for MM patients with MM had good discriminative validity in the validation dataset.

**Table 7:** Risk stratification of infection patients in the validation group.

Risk level	Risk stratification	Presence or absence of infection		Total
		Absence	Presence	
Risk level	0–2	40 (80.00)	18 (45.00)	58 (64.44)
	3–5	10 (20.00)	18 (45.00)	28 (31.11)
	6–7	0 (0.00)	4 (10.00)	4 (4.44)
total		50	40	90

**Figure 3:**
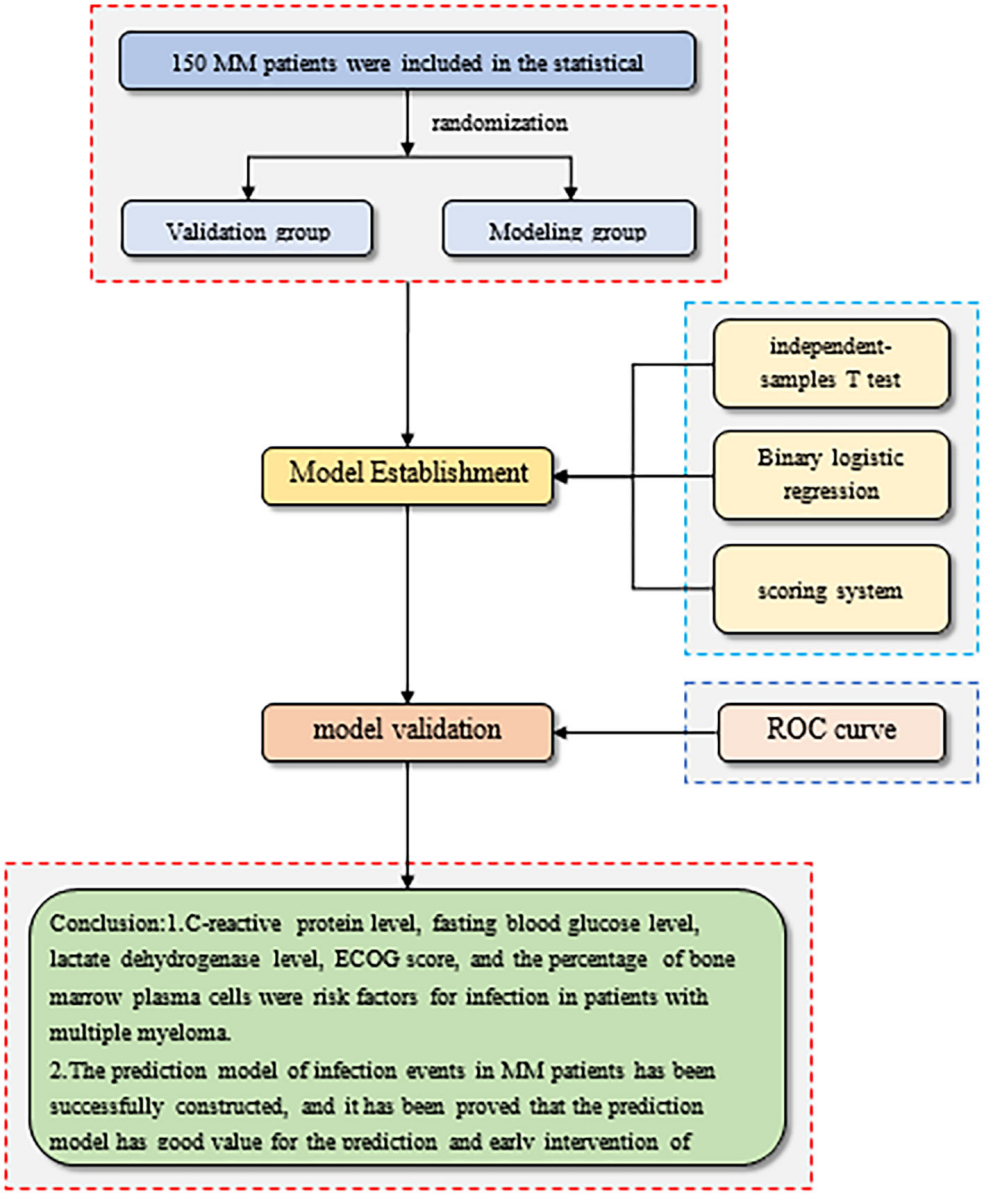
Flow chart of model construction and validation.

The flow chart of model construction and validation is shown below. The modeling flowchart is shown in **Figure 3**.

## 5 Discussion

Multiple myeloma (MM) is a malignant hematological disorder characterized by the abnormal proliferation of clonal plasma cells. It ranks as the second most prevalent hematological malignancy in numerous countries and primarily affects the elderly, accounting for approximately 2% of cancer-related mortality, particularly in the United States, Australia, and Western Europe (13–16).Due to the cumulative effects of the disease itself, treatment, and host-related factors, infection remains the leading cause of death in patients with MM (17), especially during the early period of MM treatment (5, 8, 18, 19). Blimark et al. (20)reported that MM patients with MM had a significantly higher risk of infection than the general population, with infection rates for viruses and bacteria being approximately 10-fold and 7-fold higher than normal people, respectively. In addition, studies (21, 22) also found that infection is the main cause of early death in MM patients within the first year after diagnosis. 23 analyzed all infectious events in 1,347 patients enrolled in four Grupo Español de Mieloma (GEM) clinical studies that included newly diagnosed MM patients who were transplant eligible and transplant ineligible. Albumin ≤30 g/L (OR 2.12, p < 0.001), ECOG > 1 (OR 1.73, p = 0.005), male sex (OR 1.50, p = 0.037), and non-IgA MM (OR 1.49, p = 0.091) were identified as risk factors for early severe infection. Based on these variables, a scoring system was generated to predict the risk of infection (GEM-PETHEMA) was generated, dividing patients into three groups: low-risk group (0–2 points), intermediate-risk group (3–4 points), and high-risk group (4–5 points) groups. The probability of early severe infection in these groups was 8.2%, 19.2%, and 28.3%, respectively. 24 found that an ECOG score > 2, β2-microglobulin > 6 mg/L, lactate dehydrogenase > 200 U/L, and hemoglobin < 11 g/dL were significantly associated with infection in patients with MM. These findings partially align with the present study.

This study identified several risk factors associated with infection in multiple myeloma (MM) patients with MM, including a C-reactive protein level greater than 10 mg/L, a fasting blood glucose level exceeding 6.1 mmol/L, a lactate dehydrogenase level of 245 U/L or higher, a bone marrow plasma cell percentage of 30% or more, and an Eastern Cooperative Oncology Group (ECOG) score greater than 3. Studies have reported (25–27) that elevated CRP levels in the blood are closely related to poor prognosis in MM, largely due to the increased production of inflammatory cytokines such as tumor necrosis factor (TNF) and interleukin-1 (IL-1) induced by MM cells. However, CRP may also act directly on myeloma cells to promote their proliferation under stress, leading to further weakening immune function and increasing infection risk. When the glucose level is higher than the normal value, it mainly affects the chemotaxis, migration, and phagocytosis of neutrophils, which significantly reduces their sterilization ability (28). It is well established that the current standard first-line treatment for multiple myeloma (MM) is the combination of bortezomib, lenalidomide, and dexamethasone, commonly referred to as “VRD.” Notably, hyperglycemia induced by dexamethasone can compromise the immune system of MM patients, facilitating the colonization of pathogenic bacteria and increasing the likelihood of infections. Immunodeficiency is a significant characteristic of multiple myeloma (MM) patients. A higher percentage of bone marrow plasma cells correlates with an increased tumor burden, which subsequently leads to a further decrease in immunoglobulin levels, thereby exacerbating the risk of infection risk. In light of these risk factors, this study developed a predictive model that effectively forecasts infection events in MM patients. Additionally, risk stratification was performed based on the scores, facilitating the identification of high-risk patients. The receiver operating characteristic (ROC) curve was utilized to evaluate the model, demonstrating that the infection risk prediction scoring system for MM patients possesses robust discriminative validity in both the modeling and validation groups. This model will assist clinicians in assessing the infection risk in MM patients with MM. To enhance the effectiveness of anti-infection treatment in patients with multiple myeloma (MM, it is essential to implement tailored prevention and treatment strategies based on varying infection risk levels among these patients.

Multiple myeloma (MM) is a type of blood cancer characterized by the malignant proliferation of plasma cells, mainly occurring in the bone marrow. The tumor microenvironment (TME) plays a crucial role in the disease’s progression, drug resistance, and treatment response (29). The following provides a detailed discussion of the tumor microenvironment of MM.

1. Interaction of bone marrow stromal cells. The interaction between MM cells and bone marrow stromal cells is complex. This interaction not only supports the survival of tumor cell survival but also promotes their proliferation and the development of drug resistance. Bone marrow stromal cells secrete cytokines and growth factors to form a protective microenvironment that helps tumor cells evade treatment effects of drugs (30).
2. Environmentally-mediated drug resistance (EMDR). Environment-mediated drug resistance is a form of drug tolerance, in which the bone marrow microenvironment protects tumor cells from the effects of treatment through multiple mechanisms. This protective effect may occur by enhancing cell survival signaling pathways and inhibiting cell death signaling pathways (31).
3. Cell adhesion-mediated drug resistance (CAM-DR). In hematological malignancies, cell adhesion-mediated drug resistance is particularly significant. MM cells can adhere to bone marrow stromal cells, a process that facilitates the survival and promotes resistance to develop drug resistance to chemotherapy (32). This mechanism affects drug efficacy of drugs by regulating intracellular signaling and cell cycle changes (33).
4. Internal and external mechanisms. Drug resistance in MM arises through a combination of intrinsic and extrinsic mechanisms. Intrinsic mechanisms are typically related to genetic mutations or expression changes within the tumor cells, whereas extrinsic mechanisms primarily involve the tumor microenvironment. For instance, tumor cells may increase their survival capabilities through genetic mutations or epigenetic alterations.
5. Metabolic reprogramming. Metabolic reprogramming is an important strategy that MM cells use to adapt to microenvironmental changes in the microenvironment and drug stress. Tumor cells can enhance their tolerance to therapy by modifying metabolic pathways, such as increasing the production of certain metabolites that counteract the toxicity of chemotherapy toxicity.
6. Regulation of microRNA. MicroRNAs (miRNAs) also play a significant role in drug resistance in MM. These small RNA molecules can influence tumor cell survival and proliferation of tumor cells by regulating the expression of specific target genes. Changes in miRNA expression—either upregulation or downregulation—may affect tumor response of tumor cells to therapy.
7. PD-1/PD-L1 pathway. Immune escape is an important characteristic of MM cells. The upregulation of the PD-1/PD-L1 pathway allows tumor cells to suppress the immune responses, enhancing their survival ability and contributing to treatment resistance. This mechanism not only affects both tumor cells and immune cells within the microenvironment.

In conclusion, this study retrospectively analyzed the clinical data of 150 multiple myeloma (MM) patients and identified infection-related risk factors for infections in this population using a regression model. A predictive model for infection events in MM patients was successfully developed, demonstrating significant value for prediction and facilitating early intervention. Currently, there are limited studies, both domestically and internationally, focusing on the prediction of infection events in MM patients.

In this study, a total of 44 MM patients experienced infection events (44/180, 29.3%). Pulmonary infection, upper respiratory tract infection, urinary tract infection, bloodstream infection, and herpes zoster virus infection occurred in patients with MM during chemotherapy, among which the incidence of pulmonary infection and urinary tract infection was relatively high. Very few patients had multiple simultaneous infections (pulmonary infection + bloodstream infection + herpes zoster virus infection). In this study, we successfully constructed a prediction model for MM infection events, demonstrating its value in forecasting such events. However, this study has certain limitations. First, the total number of cases analyzed was only 150, resulting in a relatively small sample size that may introduce bias. Second, the data used to construct and validate the prediction model were sourced exclusively sourced from our own hospital, lacking multi-center research and external data validation. In future studies, we plan to expand the patient sample size to reduce bias and conduct more multi-center, prospective research to provide further validation.

## Data Availability

The datasets presented in this study can be found in online repositories. The names of the repository/repositories and accession number(s) can be found in the article/Supplementary Material.
